# Whole transcriptome sequencing identifies tumor-specific mutations in human oral squamous cell carcinoma

**DOI:** 10.1186/1755-8794-6-28

**Published:** 2013-09-04

**Authors:** Qu Zhang, Jun Zhang, Hong Jin, Sitong Sheng

**Affiliations:** 1 Department of Human Evolutionary Biology, Harvard University, Cambridge, MA 02138, USA; 2 Department of Surgery, Shanghai Institute of Digestive Surgery, Rui Jin Hospital, Shanghai Jiaotong University, School of Medicine, No.197 Ruijin er Road, Shanghai, P.R. China; 3 HYK High-throughput Biotechnology Institute, 4th Floor, Building No.11, Software Park, 2nd Central Keji Rd, Hi-Tech Industrial Park, Shenzhen, China

**Keywords:** RNA-Seq, Oral squamous cell carcinoma, Somatic mutations, Significantly mutated genes, Differential expression, Disruptive genes

## Abstract

**Background:**

The accumulation of somatic mutations in genes and molecular pathways is a major factor in the evolution of oral squamous cell carcinoma (OSCC), which sparks studies to identify somatic mutations with clinical potentials. Recently, massively parallel sequencing technique has started to revolutionize biomedical studies, due to the rapid increase in its throughput and drop in cost. Hence sequencing of whole transcriptome (RNA-Seq) becomes a superior approach in cancer studies, which enables the detection of somatic mutations and accurate measurement of gene expression simultaneously.

**Methods:**

We used RNA-Seq data from tumor and matched normal samples to investigate somatic mutation spectrum in OSCC.

**Results:**

By applying a sophisticated bioinformatic pipeline, we interrogated two tumor samples and their matched normal tissues and identified 70,472 tumor somatic mutations in protein-coding regions. We further identified 515 significantly mutated genes (SMGs) and 156 tumor-specific disruptive genes (TDGs), with six genes in both sets, including *ANKRA2*, *GTF2H5*, *STOML1*, *NUP37*, *PPP1R26*, and *TAF1L*. Pathway analysis suggested that SMGs were enriched in cell adhesion pathways, which are frequently indicated in tumor development. We also found that SMGs tend to be differentially expressed between tumors and normal tissues, implying a regulatory role of accumulation of genetic aberrations in these genes.

**Conclusions:**

Our finding of known tumor genes proves of the utility of RNA-Seq in mutation screening, and functional analysis of genes detected here would help understand the molecular mechanism of OSCC.

## Background

Squamous cell carcinoma is one of the most commonly observed cancers worldwide [1], which is often diagnosed in the oropharynx and oral cavity. It is highly invasive and metastatic at the advanced stage, and presents a substantial threat to human health [2]. Evidence from various molecular and genetic studies suggests an association between squamous cell carcinoma initiation and development and the accumulation of genetic alterations at both the DNA and RNA levels [3]. Genomic alterations such as point mutations and copy number variations, epigenetic changes such as methylation and histone modifications, as well as gene expression changes have been previously revealed in oral squamous cell carcinoma (OSCC), which could facilitate biomarker development and make clinical decisions [3]. Among them, mutations only occurring in tumor tissues, often referred as somatic mutations, are given particular attention. It is widely accepted that tumors develop through the accumulation of somatic mutations in specific genes, depending on their types [4]. Various studies have found a higher than expected mutation frequency of candidate cancer genes, and that the tumor properties could be influenced by different combinations of mutations [5-8]. However, the high cost of Sanger sequencing prevents global profiling of somatic mutations in OSCC, and further understanding in mechanisms and clinical treatments.

Remarkable advances in sequencing technology over the last several years make possible to identify genetic alterations in a genome-wide scale. RNA-Seq is a newly developed deep sequencing technology, which is extensively applied in transcriptomic profiling due to its affordable cost. Compared with long standing methods such as microarray, RNA-Seq gives a far more precise measurement of transcript expression levels and a far more sophisticated characterization of transcript isoforms [9,10]. Therefore it has been successfully applied to identify differentially expressed genes [11] and to characterize allele-specific expression patterns [12,13]. Moreover, it is also an efficient and cost-effective way to study genomic alterations, such as somatic mutations in transcribed regions [14-17] or gene fusions [12-14]. Herein, we conducted a genome-wide study to investigate the somatic mutation spectrum in OSCC by interrogating RNA-Seq data from two tumor samples and their matched normal samples. We developed a sophisticated pipeline to identify somatic mutations, and then identified significantly mutated genes (SMG) and tumor-specific disruptive genes (TDG). By comparing with gene expression pattern, we also found a correlation between differentially expressed genes and SMGs. These findings demonstrate the ability of RNA-Seq to characterize global pattern of somatic mutations and suggest the potential mechanism on how somatic mutations could affect tumor development.

## Methods

### Deep sequencing data

Whole transcriptome short reads of three paired tumor and normal tissues from patients with oral squamous cell carcinoma (OSCC) were downloaded from European Nucleotide Archive (ENA, http://www.ebi.ac.uk/ena) with the accession number SRP002009. As described in the original paper, the study was conducted according to the Declaration of Helsinki, and was approved by the Institutional Review Board of the Mayo Clinic [18]. Written informed consent for the collection of samples and subsequent analysis was available for all patients. 50-bp sequence reads were generated by using Applied Biosystems SOLiD System (V3 chemistry), following the manufacturer’s instructions. More details can be found in the original study [18].

### Sequence alignments

We first excluded low quality reads in which one or a few bases have Q-score lower than 20. The initial quality check suggests that all reads from one patient have an average quality score < 20 and were excluded from further analysis. Then qualified short reads were aligned to 18,462 transcripts of UCSC consensus coding sequences (CCDS) in current human genome assembly (hg19, http://genome.ucsc.edu/). The alignment was carried out using BFAST [19], using options for color-space reads.

### Variant calling and identification of somatic variants

**Figure 1:**
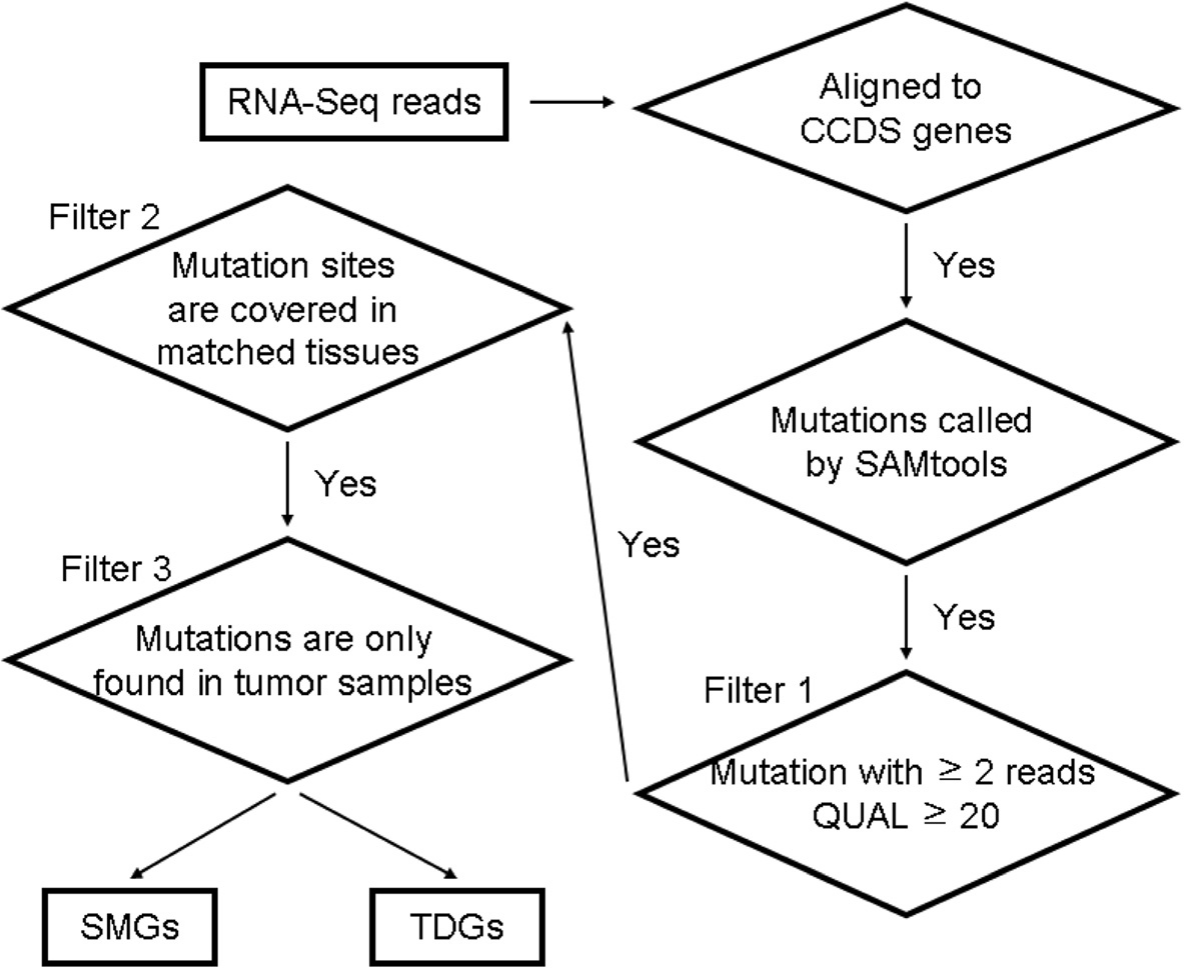
**Flowchart of the bioinformatics pipeline.** The input data is high-quality reads in which each base has a Q-score ≥ 20. The output file is somatic mutations in tumor samples and was further feed into pipelines to identify significantly mutated genes (SMGs) and tumor-specific disruptive genes (TDGs). See Methods for more details.

We called variants from the SAM format read alignments using SAMtools package [20] for each sample. We first discarded alignments with the mapping quality lower than 30, and then made variant calls by mpileup and bcftools programs embedded in SAMtools [20]. To avoid potential PCR duplicate fragments, we discarded all variants covered by more than 500 reads, which was achieved by setting –D as 500 when invoking vcfutils.pl script. Several additional filters were also applied to minimize false positive rate (Figure 1):

Filter 1 Variants were removed if they are supported by less than two reads or mistakenly called with a probability greater than 0.01. This was done by requiring a value ≥ 20 for the ‘QUAL’ column in vcf files generated by SAMtools.

Filter 2 Somatic variants were called by comparing matched normal and tumor tissues. We first excluded variants located in genomic regions of poor quality, which were defined as regions with read coverage in only one of a sample pair, probably due to randomness in sequencing process.

Filter 3 Variants that are found in both of the matched normal and tumor samples were discarded. Also, variants found in dbSNP build 132 [21] were also excluded.

### Identification of significantly mutated genes

To find significantly mutated genes, we adopted approaches implemented in MuSiC analysis tool suites [22]. Briefly, we counted the number of bases with at least three read depth in six categories, including A bases, T bases, C bases in CpG, G bases in CpG, C bases not in CpG, and G bases not in CpG. Then the discovered mutations were categorized as AT transitions, AT transversions, CpG transitions, CpG transversions, CG (non-CpG) transitions, CG transversions, and indels. Next, we calculated the background mutation rate (BMR) for each mutation category, which was done by dividing the total count of such category by the total number of available bases within such category. For indels, BMR is calculated as the total number of bases covered by indels divided by the total number of high quality bases. Since we used RNA-Seq data and the read depth depends on the expression level, tests that consider mutation coverage to identify significantly mutated genes may not be appropriate due to confounding factors such as allele-specific expression. Instead, we calculated the possibility of finding more mutations than the observation for each mutation category and combined then by the simple Fisher’s combined *P*-value test (FCPT) to generate a statistic [22], and the final *P*-value can be calculated according to a *χ*^2^ distribution with two times the number of categories as the degrees of freedom.

### Functional and pathway enrichment analysis

To identify enriched gene functions, we extracted functional annotations from gene ontology (GO) [23] using bioconductor (http://www.bioconductor.org) package “org.Hs.eg.db”. Then we used “topGO” package in R software [24] to perform hypergeometric tests. Kyoto Encyclopedia of Genes and Genomes pathway information [25,26] was extracted using bioconductor package “KEGG.db”, and hypergeometric tests were used to identify enriched pathways.

### Differential gene expression

To estimate gene expression abundance, we counted the number of reads that were aligned to each gene transcript. We then used bioconductor package “DESeq” [27] to identify differentially expressed genes. DESeq assumes a negative binomial distribution to estimate variance and mean for each group, and performs statistical test based on it. Multiple-testing was corrected by Benjamini and Hochberg procedure [28].

## Results

### Read alignments and mutation spectrum

Transcriptomes of paired normal and tumor tissues from two OSCC patients were sequenced by Applied Biosystems SOLiD System, and ~1,200 million short reads were generated (200 million reads per sample), each 50-bp long [18]. A total of 187 million reads passed the filter and were aligned to 18,462 transcripts of UCSC CCDS genes using BFAST aligner (Table 1). To minimize possible sources of false positive in variant calling, we only kept ~23 million aligned reads (12.4%) in total with a minimal mapping quality score of 30. Then we used programs in SAMtools package to call variants. Due to the high error rate in massively parallel sequencing technique and short read alignment, we took extra care in variant identification and applied a series of stringent filters. Finally, we identified 144,400 somatic mutations in all samples and 70,472 of them passed all three filters (Table 2), and both patients showed a significant excess of somatic mutations in tumors (*P* < 2 × 10^-16^ for patient 33 and *P* = 5.6 × 10^-6^ for patient 51, chi-square test), which is expected.

**Table 1:** Summary statistics of whole transcriptome sequencing data used in this study

	**Patient 33**		**Patient 51**	
	**Normal**	**Tumor**	**Normal**	**Tumor**
Total reads	229 M	256 M	227 M	199 M
HQ reads (%)^a^	68.9 M (30.0)	47.0 M (18.3)	53.1 M (23.4)	17.8 M (9.0)
HQ mapped (%)^b^	6.4 M (9.3)	4.4 M (9.3)	9.1 M (17.1)	3.4 M (18.8)

^a^*HQ reads* high-quality reads in which each base has a Q-score ≥ 20. Numbers in the brackets are percentages of high quality reads out of total reads.

^b^*HQ mapped* number of high quality reads of which mapping quality score ≥ 30. Numbers in brackets are percentages of those mapped reads out of high quality reads.

**Table 2:** Summary statistics of variants or genes after each bioinformatic filter

	**Patient 33**		**Patient 51**		**Sum**
	**Normal**	**Tumor**	**Normal**	**Tumor**	
Raw	44,185	41,645	41,970	16,600	144,400
After filter 1	33,112	31,285	34,164	13,020	111,581
After filter 2	23,869	24,950	24,636	11,811	85,266
After filter 3	20,175	21,246	20,861	8,190	70,472
Coding	9,367	10,295	8,870	3,476	32,008
Disruptive	8,058	8,827	7,590	2,835	27,310
Disruptive genes	4,454	4,758	4,404	2,076	15,692

### Significantly mutated genes

One distinguishable feature of tumor driver genes is the unexpectedly high somatic mutation rate, which leads to rapid accumulation of genetic aberrations and thus radical modification or disruption of gene functions. In hopes of finding tumor driver genes, we adopted an approach developed elsewhere [22] to identify significantly mutated genes (SMGs). Since the number of sequencing reads is highly variable among samples, we applied the pipeline to each sample separately. Significantly mutated genes in tumors were defined as genes that have ≥ 100 base pairs covered by least three reads and have a FCPT *P*-value < 0.01 in the two tumor samples but not in neither of the normal samples. In total, 515 significantly mutated genes were identified among 11,065 genes expressed in all samples (Additional file 1), and their average mutation rate (0.0018 per base) is significantly higher than that of other genes (0.0008 per base, *P* = 2.89 × 10^-15^).

### Genes with disruptive mutations

Genes with disruptive mutations in tumor samples are also of great interest, as they embrace the potential to radically change gene functions. To identify disruptive mutations, we annotated 70,472 somatic mutations identified above and searched for nonsynonymous mutations and indels. In total, 27,310 disruptive mutations were found in all samples (Table 2). Since our purpose was to identify tumor-specific disruptions, we only focused on tumor-specific disruptive genes (TDGs) which contain disruptive mutations in the two tumor samples but not in any normal samples. As a result, 156 genes were found as TDGs, of which six genes were also identified as SMGs.

### Gene ontology and pathway analysis

We further performed gene ontology and pathway analysis on both SMG and TDG sets. Although no functional categories were enriched in TDGs, we found several enriched GO terms in SMGs (Table 3), including voltage-gated cation channel activity, intrinsic to membrane, integral to membrane, intrinsic to plasma membrane and integral to plasma membrane. By interrogating KEGG pathways, we found SMGs were highly overrepresented in neuroactive ligand-receptor interaction, cell adhesion molecules (CAMs), and complement and coagulation cascades, while TDGs were enriched in steroid biosynthesis, ribosome, and aldosterone-regulated sodium reabsorption, at a relaxed *P*-value (0.1). The difference in functions and pathways between SMGs and TDGs suggests we captured different features of tumor in oral squamous cell carcinoma.

**Table 3:** Enriched GO and pathway categories

**Term**	**Description**	**Adjusted *P***	**Category**^**a**^	**GeneSet**^**b**^
GO:0022843	voltage-gated cation channel activity	0.019	MF	SMG
GO:0031224	intrinsic to membrane	0.001	CC	SMG
GO:0016021	integral to membrane	0.001	CC	SMG
GO:0005887	integral to plasma membrane	0.009	CC	SMG
GO:0031226	intrinsic to plasma membrane	0.011	CC	SMG
HSA04080	Neuroactive ligand-receptor interaction	0.055	KEGG	SMG
HSA04514	Cell adhesion molecules (CAMs)	0.052	KEGG	SMG
HSA04610	Complement and coagulation cascades	0.052	KEGG	SMG
HSA00100	Steroid biosynthesis	0.110	KEGG	TDG
HSA03010	Ribosome	0.110	KEGG	TDG
HSA04960	Aldosterone-regulated sodium reabsorption	0.110	KEGG	TDG

^a^*MF* molecular function term in GO, *CC* cellular component term in GO, *KEGG* KEGG pathway terms.

^b^*SMG* significantly mutated genes, *TDG* tumor-specific disruptive genes (see main text for details).

### Somatic mutations and gene expression

To understand the potential consequence of SMGs and TDGs, especially in gene expression, we estimated gene expression abundance as the number of high-quality reads mapped to each gene, and used “DESeq” to identify genes with significantly differential expression between tumors and normal samples. In total, we found 41 differentially expressed genes (DEGs) with an adjusted *P* < 0.05. Among them, five genes are SMGs, and one is TDG. The number of shared genes between DEGs and SMGs was highly unexpected (*P* = 0.002, hypergeometric test), while no such pattern was observed for TDGs (*P* = 0.07), indicating that SMGs may function through transcriptional regulation.

### Functional consequence of candidate genes

**Figure 2:**
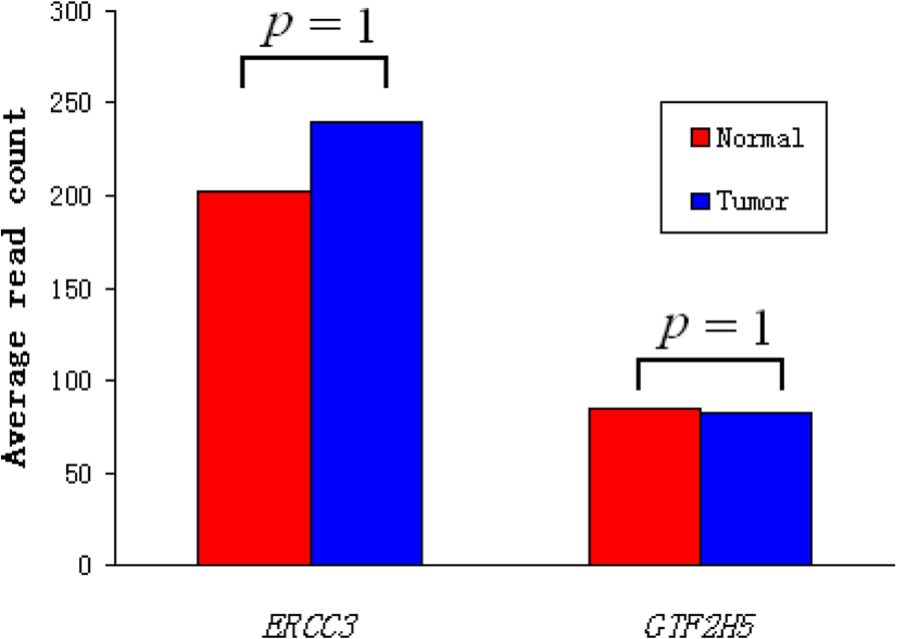
**Gene expression pattern for *GTF2H5 *and its downstream gene *ERCC3*.** The expression level was estimated by RSEM, which counts the number of reads mapped to each transcript and normalized by the total number of reads in each sample. No significant difference was found between tumor and normal tissues for each gene.

There are six genes (*TAF1L*, *ANKRA2*, *STOML1*, *PPP1R26*, *NUP37*, and *GTF2H5*) identified in both SMGs and TDGs, which constitute the prioritized candidates for detailed functional dissection. Of them, five (*TAF1L*, *ANKRA2*, *STOML1*, *PPP1R26*, and *NUP37*) were reported in the Catalogue Of Somatic Mutations In Cancer (COSMIC, http://www.sanger.ac.uk/genetics/CGP/cosmic/, v62 release). However, somatic mutations in *GTF2H5* identified in this study were not observed in 90 analyzed samples of the database. *GTF2H5* encodes a 71-aa peptide, which is a subunit of the basal transcription factor *TFIIH* and functions in nucleotide excision repair and transcription [29]. To better understand the potential role of *GTF2H5*, we first examined its expression pattern (Figure 2), but no significant difference was observed between tumor and normal tissues (*P*-value = 1 after Benjamini-Hochberg correction), nor was its downstream gene *ERCC3* (*P*-value = 1 after Benjamini-Hochberg correction). We then predicted the effect of the amino acid changes in *GTF2H5* using SIFT tool [30], however, no intolerant changes were found among these disruptive mutations. Since the major determinant in oral cancers is the accumulation of genomic instability [31], and no obvious evidence was observed in radical mutations or gene expression, it is possible that the excess of somatic mutations in *GTF2H5* may influence post-transcriptional regulation and correlate with tumor development.

## Discussion

It is well accepted that the accumulation of multiple genetic events in different genes and molecular pathways is the main cause of OSCC evolution [3,32]. Previous studies have identified various types of genetic aberrations in OSCCs and oral dysplasias, the precursors of OSCCs, including somatic mutations in the D-loop of mtDNA sequence [33] and in exons nine and 20 of Phosphatidylinositol 3-kinase gene (*PI3K*) [34], common deletions on chromosome 3p such as the 3p14 locus that harbors *FHIT* (Fragile Histidine Triad) [35-37], as well as gene copy number increases in certain oncogenes such as *EGFR* and *CCND1*[31,38,39]. Evidence from microarray studies has also revealed differentially expressed genes in oral cavity tumors [40-44], suggesting multiple dimensions of genetic aberrations contributing to OSCC development. Here, we presented a whole transcriptome analysis to identify exonic somatic mutations in two OSCC samples. To overcome the small sample size, we have developed a stringent bioinformatic pipeline with multiple filters to reduce false positives. In total, we have identified 515 SMGs which were significantly mutated, and 156 TDGs with disruptive mutations in both tumor samples. We also measured gene expression and found SMGs were enriched in differentially expressed genes, implying that the accumulation of genetic aberrations may regulate corresponding gene expression and further affect tumor evolution.

Five of six genes identified in both SMGs and TDGs are known driver genes in COSMIC database, and the remaining gene *GTF2H5* stimulates the ATPase activity of *ERCC3*, a nucleotide excision repair gene, to trigger DNA opening during DNA repair. Since genes involved in DNA repair functions are commonly associated with oral cancer [45-47], it is very likely that *GTF2H5* is also related to carcinogenesis. Collectively, these observations indicate our bioinformatic pipeline has substantial power to identify tumor-related genes.

Of 515 SMGs, several membrane-related GO terms were enriched, including intrinsic to membrane, integral to membrane, intrinsic to plasma membrane and integral to plasma membrane. Interestingly, the original study found that the term of intrinsic to plasma membrane was enriched in mis-regulated genes in tumor samples [18], suggesting that disruption or mis-expression of genes related to plasma membrane may be involved in tumor development. We also found six tumor related genes, *TUSC2*, *TP53I3*, *TSSC4*, *RAB23*, *RAB39A*, and *ERG*, which function as either tumor suppressor genes or oncogenes. Additionally, we identified *FGF2*, a fibroblast growth factor in the FGF signaling pathway, which was reported to be important in OSCCs [3]. Another pathway potentially associated with OSCC is the cell adhesion molecules (CAMs), which is also enriched in SMGs. CAMs are essential component to maintain the structure of stratified squamous epithelium and a critical mediator of tumor progression in OSCC [48,49], and mis-expression or dysfunction of CAMs are shown to contribute to malignant tumors [48]. The excess of CAMs in SMGs further suggests the critical role of the CAM pathway in OSCCs. Again, cell adhesion was found to be enriched in mis-regulated genes in the original study [18], confirming that tumor development may involve both mis-expression and dysfunction of CAMs.

Tumor driver genes are normally considered as with high somatic mutation rate, thus 156 TDGs identified without information from mutation rate are intriguing. Besides six genes also identified as SMGs, we also found that 57 (37%) TDGs significantly mutated in one tumor sample but not in the other tumor sample. Considering that only two patients were used in this study and a large proportion of TDGs were significantly mutated in only one sample, it is possible that some TDGs are in fact SMGs, but failed to be identified here due to the small sample size. We thus suggest that screening TDGs may be an alternative way to identify candidate cancer driver genes when sample size is limited.

Although a few pioneer studies demonstrated that RNA-Seq is suitable for identifying somatic mutations [14-17,50,51], there is a concern that RNA-Seq is prone to error [15] and may generate a high false discovery rate due to incorrect alignment of reads, sequencing errors or extremely high or low read coverage. To minimize the false positive rate, we have applied a series of stringent filters. First, we only used reads in which each base has a Q-score ≥ 20, which reduces the influence of sequencing errors. Next we filtered out read alignments with a mapping quality lower than 30, which avoids reads mapped to multiple locations alignments with low similarity. Then we required each qualified variant must have a read depth between three and 500. Our strategy to identify somatic mutations also automatically removed the effect of systematically incorrect alignments which present in both tumor and matched samples. Hence we believe that somatic mutations identified in this study provide a substantial list of candidates for biomarker development. However, it should also be noted that we only focused on exonic regions captured by RNA-Seq, somatic mutations in regulatory regions will not be identified here, therefore out list also presents a portion of somatic mutations in OSCCs.

## Conclusions

In this study, we have developed a stringent bioinformatic pipeline to identify somatic mutations in tumors and applied it to two OSCC paired samples. By using multiple filters and calling candidate disruptive genes through two different ways, we minimized both false positives and false negatives due to the small sample size. The resulting candidate genes with both statistical and biological significance would help understand the molecular mechanism of OSCC and develop clinical biomarkers and drug targets.

## Competing interests

The authors declare no conflict of interest.

## Authors’ contributions

QZ and SS conceived the project. QZ, JZ, and HJ carried out the data analysis. QZ and SS drafted the manuscript. All authors read and approved the final manuscript.

## Pre-publication history

The pre-publication history for this paper can be accessed here:

http://www.biomedcentral.com/1755-8794/6/28/prepub

## Supplementary Material

Additional file 1 List of significantly mutated genes (SMGs) and tumor-specific disruptive genes (TDGs). Click here for file
